# *Coelotesvignai* Brignoli, 1978 (Araneae: Agelenidae) from Turkey: first description of male and annotations on terminology of copulatory organs

**DOI:** 10.3897/BDJ.9.e73127

**Published:** 2021-10-08

**Authors:** Dragomir Kostov Dimitrov, Peter Jäger

**Affiliations:** 1 University of Barcelona, Barcelona, Spain University of Barcelona Barcelona Spain; 2 Senckenberg Research Institute, Frankfurt am Main, Germany Senckenberg Research Institute Frankfurt am Main Germany

**Keywords:** *charitonovi* group, Coelotinae, morphology, taxonomy, terminology

## Abstract

**Background:**

The agelenid spider species *Coelotesvignai* Brignoli, 1978 was described, based on female specimens from Turkey.

**New information:**

The unknown male is here described, based on specimens from the type locality: Bolu, Abant Mountains, Turkey. The variation of the female copulatory organs is illustrated. The relationships of the species with its putative closest congeners are discussed. The discrepancy between the morphological terminology used in the Coelotinae and Ageleninae is discussed and some suggestions how to unify them are proposed.

## Introduction

Brignoli (1978) described *Coelotesvignai* along with two other similar species – *C.arganoi* and *C.coenobita* from Turkey, all of them only by females. Ovtchinnikov (1999) created the subgenusBrignoliolus and included there *C.arganoi*, but not the other two species. According to him, the subgenus has an Irano-Turanian distribution, reaching the Western Tian Shan in the East. However, there are no species recorded from that area so far (World Spider Catalog 2021). Ovtchinnikov (1999) just mentioned "one more undescribed species from Western Tian Shan (Kyrgyzstan)" without providing exact locality data or any material deposition. The species in question was never described or mentioned subsequently in the literature. Wang and Zhu (2009) made a revision of the *Coelotescharitonovi* species group, including all of the *Brignoliolus* species, plus *C.coenobita* and *C.vignai*. They provide no reasons for ignoring the subgenusBrignoliolus and transformed it instead to a species group.

When sorting some Agelenidae collected from Turkey in the collection of the Senckenberg Research Institute, Frankfurt, Germany, we found four male and four female specimens of *C.vignai*, collected from the type locality. The male is described for the first time and the relationships of the species are discussed considering its closest congeners.

## Materials and methods

The material is preserved in 70% ethanol. The specimens were examined and measured using a Leica 165C stereomicroscope. All measurements are in mm. The photographs of the male palps were taken with a Canon EOS 1100D digital camera, attached to the same stereomicroscope, the ones of the habitus — with a Canon EOS R, equipped with a Canon 100 mm Macro and a Macro Ring Lite MR-14EX. Colour was described from specimens preserved in ethanol. The male palp and female vulva were dissected in order to be studied and illustrated using a Leica MZ16 stereomicroscope. The vulva was cleared in lactic acid. Leg measurements formula: total length (femur, patella, tibia, metatarsus, tarsus). Spines of legs are listed per joint in the following order: prolateral (p), dorsal (d), retrolateral (r) and ventral (v). If there is no spine, the joint is omitted; if there is more than one spine at one side of a joint, positions are listed separately for proximal, medial and distal positions, medial position might be omitted if it is a short joint. Where available, the SPD codes listed in the spider anatomy ontology (Ramirez and Michalik 2019) are implemented.

**Abbreviations: Morphology.** ALE — anterior lateral eyes, AME — anterior median eyes, AW — anterior width of prosoma, ChL — chelicerae length, ChW — chelicerae width, ClH — clypeus height (at AME), d — dorsal, LL — labium length, GL — gnathocoxae length, OL — opisthosoma length, OW — opisthosoma width, p — prolateral, bPL — prosoma length, PLA — posterior lateral eyes, PMA — posterior median eyes, PW — prosoma width, r — retrolateral, rC — retrolateral branch of conductor, SL — sternum length, SW — sternum width, TL — total length, v — ventral.

**Collections [with curators**]. NMNHS – National Museum of Natural History, Sofia, Bulgaria [S. Lazarov]; SMF – Senckenberg Research Institute, Frankfurt, Germany [P. Jäger].

## Taxon treatments

### Coelotes vignai

Brignoli, 1978

C316C8BE-E38E-581E-8710-59B82188CF54

#### Materials

- **Type status:** Other material. **Location:** continent: Asia; country: Turkey; locality: 4 males, 4 females (1 without opisthosoma), Bolu, Abant Mountains [ca. 40°36'51"N, 31°17'4"E, 1375 m a.s.l.], H. Korge leg. April 29 –May 20, 1976 (3 males, 3 females: SMF; 1 male, 1 female: NMNHS).

#### Description

**Figure 1.**
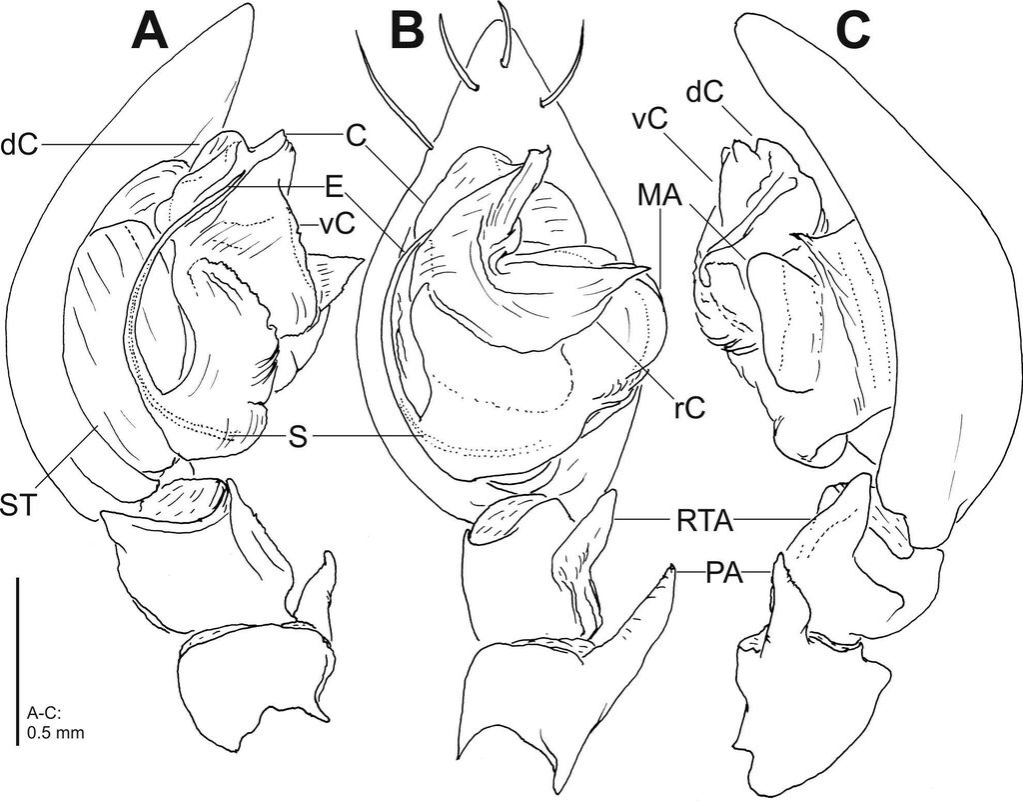
*Coelotesvignai* Brignoli, 1978, male from Bolu, Abant, Turkey, left male palp **A** prolateral; **B** ventral; **C** retrolateral views. Distal cymbial spines omitted in **A**, **C**. C—conductor [SPD:0000179], dC—dorsal part of conductor, E—embolus [SPD:0000176], MA—median apophysis [SPD:0000178], PA—patellar apophysis [SPD:0000285], rC—retrolateral branch of conductor, RTA—retrolateral tibial apophysis [SPD:0000156], S—spermophor [SPD:0000177], ST—subtegulum [SPD:0000171], vC—ventral part of conductor.

**Figure 2.**
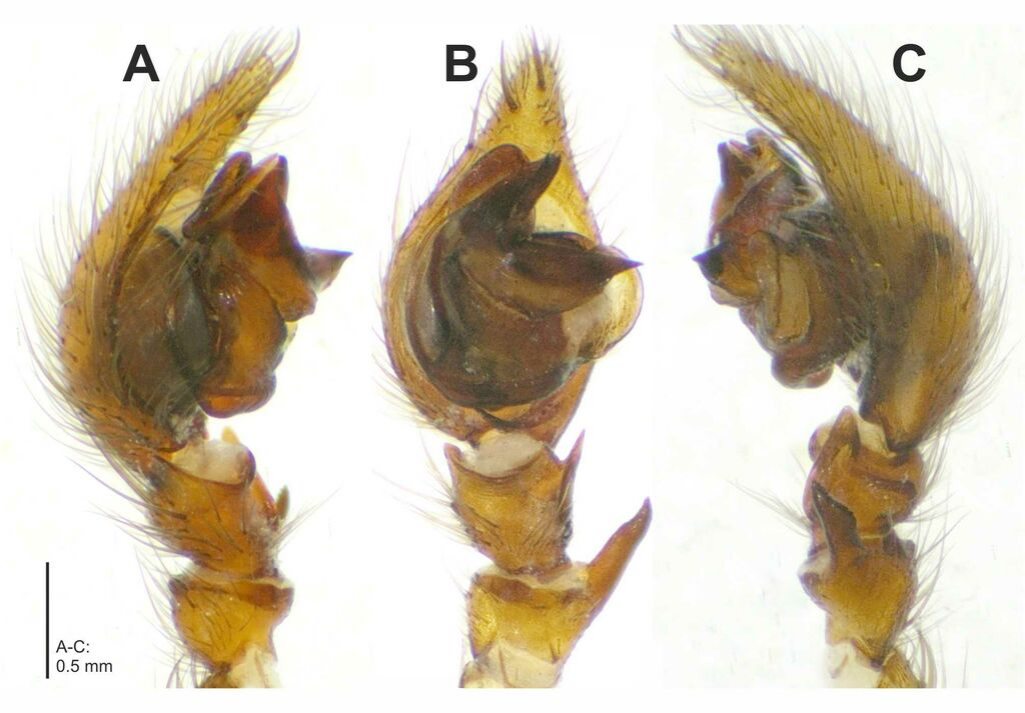
*Coelotesvignai* Brignoli, 1978. Right palp, mirrorred **A** prolateral; **B** ventral; **C** retrolateral views.

**Male** (Fig. 1A-C, Fig. 2A-C, Fig. 4A-E). TL 8.1, PL 3.9, PW 2.6, AW 1.7, OL 4.2, OW 2.5, ClH 0.21, ChL 1.98, ChW 0.83, LL 0.63, GL 1.92. Eye diameters; interdistances: AME 0.13, ALE 0.22, PME 0.15, PLE 0.18; AME-AME 0.09, AME-ALE 0.05, PME-PME 0.09, PME-PLE 0.16, AME-PME 0.07, ALE-PLE 0.03. Palp and leg measurements: palp 3.6 (1.3, 0.4, 0.3, -, 1.6), I 10.4 (2.8, 1.3, 2.5, 2.3, 1.5), II 9.7 (2.6, 1.2, 2.2, 2.2, 1.5), III 8.5 (2.3, 1.1, 1.8, 2.1, 1.2), IV 11.4 (2.9, 1.3, 2.7, 2.9, 1.6); leg formula 4123. Spination of legs: femur I p002, d110, II p001, d110, III p011, d110, r001, IV p001, d110; patella III p1, r1, IV r1; tibia I v222, II v122, III–IV p11, r11, v222; metatarsus I–II v223, III p122, r122, v2(1)22, IV p122, r1112, v222. Chelicerae with 3 promarginal and 3 retromarginal teeth, without denticles in cheliceral furrow or escort setae at base of fang. Chelicerae bulging strongly frontally (Fig. 4E: black arrow), femora I bulging slightly dorsally (Fig. 4E: white arrow).

Palp as in diagnosis (Fig. 1A-C, Fig. 2A-C). With one apophysis each on patella and tibia. Embolus thin, arising from tegulum in a 7.30-o’clock-position (Fig. 1 B, Fig. 2B). Conductor massive and complex, retrolateral branch with acute retrolaterad tip (Fig. 1B, Fig. 2B); dorsal part of the conductor well developed, forming a furrow with the ventral part, visible in retrolateral view (Fig. 1A, Fig. 1C, Fig. 2A, Fig. 2C. Median apophysis membranous, bulging retrolaterally (Fig. 1B, Fig. 2B).

Colouration (Fig. 4A-E). Deep yellowish- to reddish-brown. Prosoma with longitudinal fovea and anterior part darker, i.e. reddish-brown. Chelicerae deep reddish-brown. Other appendages yellowish- to reddish-brown, with tibiae–tarsi and femora I dorsally darker. Opisthosoma dorsally dark brown in anterior half and with distinct chevron-pattern in posterior half, laterally dotted, ventrally light greyish-brown with few dots. Spinnerets pale yellow.

**Female.** See Brignoli (1978) and Wang and Zhu (2009)

**Variation.** Males (n = 3): TL 8.0–8.6, PL 4.0–4.3, OL 4.0–4.3. Females (n = 4): TL 9.3–9.5. PL 3.6–4.2, OL 5.3. Epigynal teeth may be well separated over their entire length or fused to a certain extent. The number of the so-called “fusion bubbles” (= sclerotised spheres indicating a zone where two structures fused together, for example, median septum and lateral lobe) in the posterior part of the epigyne varies.

#### Diagnosis

**Figure 3.**
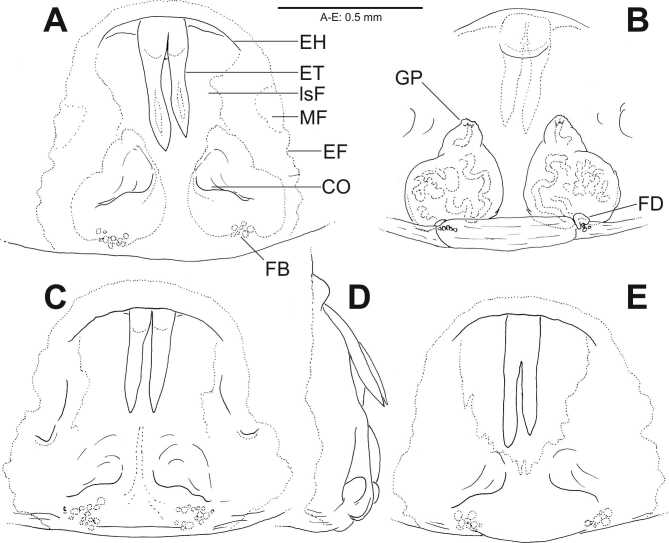
*Coelotesvignai* Brignoli, 1978, females from Bolu, Abant **A, C-E** epigyne, ventral view (A, C, E), lateral view (D); **B** vulva, dorsal. CO—copulatory opening [SPD:0000136], EF—epigynal field, EH—epigynal hood, ET—epigynal teeth, FB—“fusion bubbles”, FD—fertilisation duct [SPD:0000137], GP—glandular pores [SPD:0000139], lsF—less sclerotised field, MA—muscle attachment sigillum (**C**–**E** not dissected).

**Figure 4.**
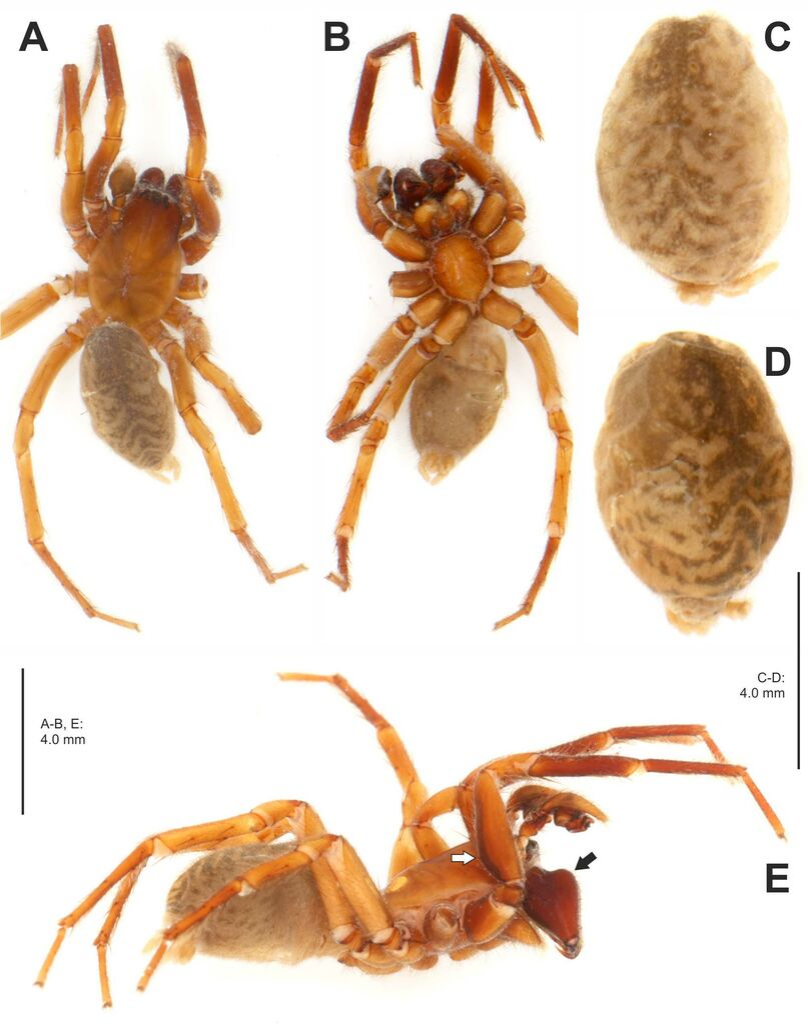
*Coelotesvignai* Brignoli, 1978 from Bolu, Abant. **A-B, E** habitus of a male: dorsal, ventral and lateral view, respectively; black arrow indicates frontal bulge of chelicerae, white arrow the dorsally bulged femur I. **C-D** opisthosomata of two females, dorsal view.

*Coelotesvignai* seems to be closely related to *Coelotescoenobita* by the shape and the position of the epigynal teeth that are long, reaching almost half of the epigynal length, situated close together with their bases situated under the epigynal hood (Fig. 3A-E; Brignoli 1978: fig. 137). *Coelotesvignai* can be distinguished from the latter by the broader epigynal hood which is not so strongly bent as in *C.coenobita* and by the copulatory openings distinct and slit-like with meandering posterior rim (Fig. 3A-C and E) where *C.coenobita* has less distinct copulatory openings with semicircular posterior rim medially (Brignoli 1978: fig. 137). The male palp differs distinctly from all the other species of *Coelotes* by the deeply notched ventral part of the conductor, forming an acute angle, with broad and proximally bulging retrolateral branch of conductor (Fig. 1A-C, Fig. 2A-C).

#### Distribution

Known only from the type locality in Turkey, Bolu Province, Abant Mountains.

## Discussion

There are some discrepancies between the terminology of the genital morphology in Coelotinae (Wang 2002, Wang and Zhu 2009) and Ageleninae (Bolzern et al. 2013). Here we propose to unify some of them. The “conductor dorsal apophysis” (sensu Wang 2002, Wang and Zhu 2009) is considered homologous to the “dorsal part of the terminal end of conductor” (sensu Bolzern et al. 2013). We find the last term more accurate since it is not actually an apophysis, i.e. not sticking out from something. Here, we simply call it “dorsal part of the conductor” and, respectively, the ventral part “ventral part of the conductor”. For the female copulatory organs, Wang and Zhu (2009) misinterpreted the terms atrium and anterior atrial margin, respectively. Atrium means cavity and is used when, from such a cavity, the copulatory ducts start, i.e. both copulatory orifices are situated in the same cavity. In *C.vignai*, there is no such cavity, neither is there one in any of the known species of this species group. What they labelled as atrium could be homologous to the “median plate – MP” sensu Bolzern et al. (2013). In addition, we prefer to call the “anterior epigynal hood” sensu Wang and Zhu (2009) simply “epigynal hood”. This term is also mentioned by Wang and Zhu (2009), but they do not make clear what is the difference between “epigynal hood” and “anterior epigynal hood” which they use interchangeably.

Morphologically, *C.vignai* and *C.coenobita* look more related to each other than to any of the other species of the *charitonovi* group by having epigynal teeth arising from under the epigynal hood. The male of *C.vignai* also differs well from the ones of the other species in the group. Although there is no information about the habitats of the two species, their restricted distribution and the fact that they inhabit the low mountain areas near the Black Sea shores imply that they are distributed in the mesophilic Euxine-Colchic broadleaf forests.

Lastly, we would like to mention the morphological similarities between the species in the *C.charitonovi* group and some other Coelotinae genera. The same type of epigynal teeth also exist in the genera *Tonsilla* Wang and Yin (1992), *Bifidocoelotes Wang (2002)* and *Draconarius* Ovtchinnikov (1999). A further revision and redefining of the Coelotinae genera may be needed in the future.

## Supplementary Material

XML Treatment for Coelotes vignai
